# Use of consumer-grade cameras to assess wheat N status and grain yield

**DOI:** 10.1371/journal.pone.0211889

**Published:** 2019-02-15

**Authors:** Enric Fernández, Gil Gorchs, Lydia Serrano

**Affiliations:** 1 Geomatics division, Centre Tecnològic de Telecomunicacions de Catalunya, Castelldefels, Barcelona, Spain; 2 Departament d’Enginyeria Agroalimentària i Biotecnologia, Universitat Politècnica de Catalunya, Castelldefels, Barcelona, Spain; College of Agricultural Sciences, UNITED STATES

## Abstract

Wheat Grain Yield (GY) and quality are particularly susceptible to nitrogen (N) fertilizer management. However, in rain-fed Mediterranean environments, crop N requirements might be variable due to the effects of water availability on crop growth. Therefore, in-season crop N status assessment is needed in order to apply N fertilizer in a cost-effective way while reducing environmental impacts. Remote sensing techniques might be useful at assessing in-season crop N status. In this study, we evaluated the capacity of vegetation indices formulated using blue (B), green (G), red (R) and near-infrared (NIR) bands obtained with a consumer-grade camera to assess wheat N status. Chlorophyll Content Index (CCI) and fractional intercepted PAR (fIPAR) were measured at three phenological stages and GY and biomass were determined at harvest. Indices formulated using RG bands and the normalized difference vegetation index (NDVI) were significantly correlated with both CCI and fIPAR at the different phenological stage (0.71 < r < 0.81, *P* < 0.01). Moreover, indices formulated using RG bands were capable at differentiating unfertilized and fertilized plots. In addition, RGB indices and NDVI were found to be related to both crop biomass and GY at harvest, particularly when data were obtained at initial grain filling stage (r > 0.80, *P* < 0.01). Finally, RGB indices and NDVI obtained with a consumer-grade camera showed comparable capacity at assessing chlorophyll content and predicting both crop biomass and GY at harvest than those obtained with a spectroradiometer. This study highlights the potential of standard and modified consumer-grade cameras at assessing canopy traits related to crop N status and GY in wheat under Mediterranean conditions.

## Introduction

Wheat (*Triticum aestivum* L) is a widely grown crop under rain-fed conditions in Mediterranean-type climate regions. Even though drought is the main constraint limiting wheat grain yield (GY) in these Mediterranean-type environments [1], nitrogen (N) fertilization has been recognized as the most cost efficient and practical tool to manage in order to optimize wheat GY [2,3]. Moreover, N availability is not only an important determinant of wheat grain yield [4,5], but adequate N supply at critical stages (i.e., at anthesis) its known to increase the qualitative appreciation of grain as a result of increased grain protein level [6]. Therefore, independently of the occurrence of water deficits, N availability is a key factor for optimizing wheat GY and quality under rain-fed conditions [7,8].

Nitrogen (N) fertilizer management is particularly challenging in Mediterranean rain-fed environments where seasonal rainfall largely dictates wheat crop N demand and GY. Indeed, water availability affects both crop growth and available soil N, making it difficult to adjust fertilizer application to crop requirements [9,10]. Under these conditions, in season diagnosis of wheat crop N status might be an effective tool to account for the effects of environmental conditions on both actual soil N supply and crop growth, enabling to adjust fertilization rates to crop demand. In addition, appropriate fertilization application might reduce the negative impacts of supplying excess N on the environment (and sustainability) [11] while improving N-use efficiency [6] as well as GY and quality. Therefore, in season diagnosis of wheat crop N status could assist producers in improving N management strategies [5].

Traditional methods to assess crop N status are based on plant analysis. For example, the well-established Nitrogen Nutrition Index (NNI) [12], requires plant N concentration and aboveground biomass as input variables. However, these methods are destructive and time consuming and are not practical for in-season diagnosis under farm conditions [13]. As an alternative to plant sampling, indirect methods using handheld optical sensors for estimating N concentration and biomass have been proposed and implemented. For example, leaf N might be estimated using leaf chlorophyll meters that relate the light transmittance to chlorophyll content [14]. Leaf chlorophyll meters are currently being applied at a commercial level in wheat crops for N fertilization management [15]. Similarly, Leaf Area Index (LAI) and green biomass might be estimated using a portable hand-held device that determines the Normalized Difference Vegetation Index (NDVI) or might be derived from sunlight interception measurements such as fractional Intercepted Photosynthetic Active Radiation (fIPAR) [16]. However, these point measurements are time-demanding and do not allow to properly characterize the spatial variability often present within fields.

Remote sensing data are an alternative to the labour intensive ground-level sampling and has proven to be a valuable tool at characterizing in-season spatial and temporal variability in crop N status [17,18]. In wheat crops, vegetation indices derived from spectral reflectance have proven useful at estimating LAI [16,19] as well as foliar N or chlorophyll content [17,20,21]. In addition, indices based on reflectance changes in the red-edge wavelengths have been developed for assessing chlorophyll content and LAI independently of variation in soil cover [20,22,23].

In the last decade, remote sensing with consumer-grade cameras has gained momentum as a low cost and easy to deploy system over both multispectral cameras and scientific-grade platforms [24]. Consumer-grade cameras are limited from factory to capture only visible light, but they can be modified and converted to a full spectrum camera—capturing from 300 to 1000 nm wavelengths [25]- by removing its internal infrared blocking filter. In addition, by using a long-pass NIR screw-in filter on top of the lens, a full spectrum consumer-grade camera might provide near-infrared (NIR) detection capabilities which are central in vegetation studies [24]. Despite some shortcomings, consumer-grade cameras -including those with NIR detection capabilities- have been gradually used in agricultural applications such as crop identification [24], LAI assessment [26], crop phenotyping [27–29] and, particularly, in monitoring crop N status [26,29–32].

Most studies have investigated the relationship between vegetation indices derived from consumer-grade cameras and biophysical parameters such as LAI and GY in wheat [27,31, among others] whereas, fewer studies have explored the relationship with N status in wheat crops [28,32–36]. The aim of this study was to evaluate the potential of vegetation indices derived from a standard (RGB) and a modified consumer-grade camera (NIR) at monitoring canopy variables related to wheat N-status. The specific objectives were: (i) to assess the relationships between vegetation indices obtained with a conventional camera and optical estimates of leaf chlorophyll content (CCI) and fractional Intercepted Photosynthetic Active Radiation (fIPAR); (ii) to determine whether these vegetation indices are reliable at detecting differences in canopy traits related to wheat crop N status, and (iii) to assess the capability of these vegetation indices at estimating wheat GY.

## Materials and methods

### Experimental site and crop management

The study was conducted at the experimental fields of the Escola Superior d’Agricultura de Barcelona (Universitat Politècnica de Catalunya) (41°16’N 1°59’E, 4.2 m a.s.l.). The area has a Mediterranean climate with a mean annual temperature of 16.5°C and an average annual rainfall of 616 mm.

Spring wheat (*Triticum aestivum* cv. Odiel) was sown in late winter (2 March 2017) in 0.15 cm wide rows at a seeding density of 425 seeds m^-2^. The experimental design was a randomized complete block with four N treatments per block and three replications. Plot size was 10 by 1.2 m. Nitrogen fertilizer rates ranged from 0 to 180 kg·N·ha^−1^, with 60 kg·N·ha^−1^ increases. Nitrogen fertilizer (i.e., ammonium nitrate) was hand broadcast in two stages: half at tillering (Zadoks code 21) [37] and half at stem elongation (Zadoks code 31). Aboveground biomass and GY were determined at maturity (29 June 2017). Three 1.2 x 0.24 m^2^ strips were hand harvested at each plot and separated into plant components (grain and aboveground biomass), dried in a 105°C oven and weighed. Grain yield was determined at 14% humidity.

### Field data collection

Field measurements were made on 17 and 28 May 2017 and on 8 June 2017. These dates corresponded to the phenological stages of anthesis (Zadoks code 65), and early and late grain filling (Zadoks code 71 and 77, respectively).

Fractional intercepted PAR was determined by measuring incident PAR above the canopy (PAR_above_) and below the canopy (PAR_below_) at five locations per plot with an AccuPAR Ceptometer (Decagon Devices, Pullman, Washington, USA) and calculated as:

$$\text{fIPAR}=\frac{\left({\text{PAR}}_{above}-{\text{PAR}}_{below}\right)}{{\text{PAR}}_{above}}$$

A CCM-200 leaf chlorophyll meter (Opti-Sciences, Hudson, New Hampshire, USA) was used to monitor crop N status. The CCM-200 chlorophyll meter is a leaf clip sensor that measures the light transmitted at two different wavebands (653 nm and 931 nm). The sensing surface is 0.71 mm2. The instrument processes the ratio of the light transmitted at these wavelengths and the ratio determined in the absence of a sample to produce a digital reading (Chlorophyll Content Index, CCI) that is highly correlated with leaf chlorophyll content [38]. Measurements were taken at the leaf longitudinal centre, on the upper side and avoiding midribs. Eight chlorophyll-meter values (CCI) were measured on the top-most fully expanded leaves and averaged for each plot.

Images were acquired in cloudless days using a modified full-spectrum consumer-grade digital camera Sony NEX-5N (Sony Corporation) mounted on a tripod boom and held in a nadir direction. The camera was positioned about 50 cm above the canopy while focusing near the centre of each plot. The camera specifications are the following: sensor Exmor APS-C CMOS 8 bit (3 channel), 16.1 megapixels (4912 x 3264), 23.5 x 15.6 mm sensor and E16 mm F2.8 fixed lens. On each plot a pair of pictures were acquired, one in standard RGB colour, and one with a modified camera fitted with a 900 nm long-pass infrared filter (Spencer’s Camera & Photo, Alpine, UT, USA). Images were acquired with a fixed ISO value 100 and a 1/1250 s shutter speed, and white balance model set to daylight conditions. These settings were chosen based on previous trials that ensured no saturation and the best dynamic range possible in terms of digital numbers for each channel. Images were recorded in RAW data format (Sony RAW v2.2) and subsequently converted to the tagged image file format (TIFF without data compression) and in standard RGB colour space using a commercial software (Sony Data Converter).

**Fig 1:**
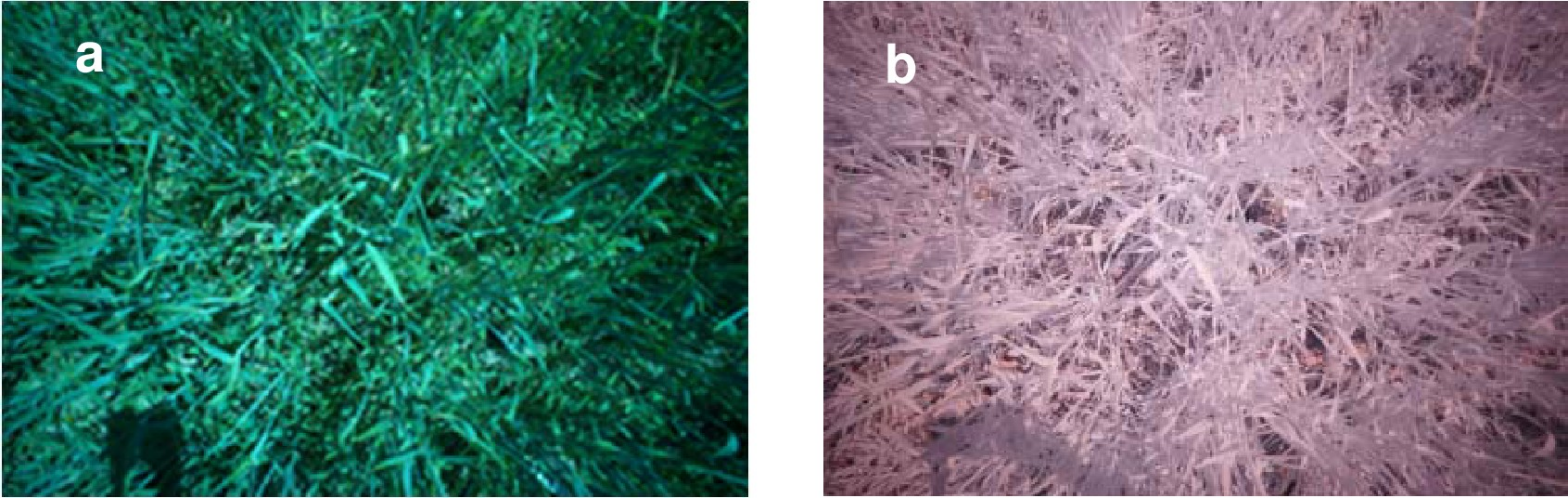
Example of images acquired at early grain filling (a) using a standard RGB camera and (b) using a modified camera fitted with a near-infrared band-pass filter.

To correct the effect of brightness reduction due to the camera optics (i.e. vignetting) [34], which is clearly noticeable in Fig 1, a circular area traced from the image centre to the borders was selected using Photoshop CS6. Subsequently, the RGB and NIR minimum, maximum and average DN values were extracted from the vignette-corrected images using image processing software (QGIS 2.14). Reflectance values were computed for each band by dividing the average DN values by those derived from images acquired on a calibrated diffuse Spectralon reflectance target (Labsphere, North Sutton, NH, USA). The reflectance target was positioned adjacent to the plots at a height above the canopy and levelled. Reflectance target images were acquired before and after canopy (plot) measurements. Several RGB-NIR vegetation indices were calculated using the reflectance data (Table 1).

**Table 1:** Summary of vegetation indices derived from red (R), green (G), blue (B) and near infrared (NIR) bands acquired with a consumer-grade camera used in this study.

Bands	Name	Acronym	Formulation	Reference
NIR / R	Normalized Difference Vegetation Index	NDVI	$\frac{\text{NIR}—\text{R}}{\text{NIR + R}}$	[39]
	Simple Ratio	SR	$\frac{\text{NIR}}{\text{R}}$	[40]
NIR / G	Green Normalized Difference Vegetation Index	GNDVI	$\frac{\text{NIR}—\text{G}}{\text{NIR + G}}$	[41]
	Green Simple Ratio	GSR	$\frac{\text{NIR}}{\text{G}}$	This study
B / R	Blue-Red Simple Ratio	BR	$\frac{\text{B}}{\;\text{R}}$	This study
B / G	Green-Blue Simple Ratio	GB	$\frac{\text{G}}{\;\text{B}\;}$	This study
G / R	Green-Red Simple Ratio	GR	$\frac{\text{G}}{\;\text{R}}$	[42]
	Normalized Difference Vegetation Index-Green	NDVIg	$\frac{\text{(G}—\text{R)}}{\;\text{(G + R)}\;}$	[43]
	Soil Adjusted Vegetation Index—Green	SAVIg	$\frac{\text{(1 + 0.5) * (G}—\text{R)}}{\text{(G + R) + 0.5}}$	[32]
	Optimized Soil Adjusted Vegetation Index—Green	OSAVIg	$\frac{\;\text{1.5 * (G}—\text{R)}\;}{\text{ (G + R) + 0.16}}$	[44]
R / G / B	Visible Atmospherically Resistant Index	VARI	$\frac{\text{(G}—\text{R)}}{\text{(G + R)}—\text{B}}$	[43]
	Red-Green-Blue Normalized Difference Vegetation Index	NDVIrgb	$\frac{\text{(G + B)}—\text{R}}{\text{(G + B) + R}}$	[44]
	Red-Green-Blue Simple Ratio	SRrgb	$\frac{\text{(G + B)}}{\text{R}}$	This study

Additionally, at the stage of late grain filling, canopy radiance was measured on each plot with a narrow-bandwidth visible and near-infrared spectroradiometer UNISPEC (PP Systems Ltd., Havervill MA, USA) with a 2.3 mm diameter bifurcated fibre optic (model UNI410, PP Systems, Havervill, MA, USA) fitted with a 12º field of view foreoptics (UNI-710, PP Systems Ltd., Havervill, MA, USA). Measurements were expressed as reflectance after standardizing by the irradiance determined using a Spectralon white reference panel. Data were collected between 11:00 h and 13:00 h (i.e., solar noon) in order to minimize disturbances from the atmosphere and changes in solar elevation. The radiometer was mounted on a tripod boom and held in a nadir orientation 0.50 m above the canopy. Four scans were internally averaged for each plot. For comparative purposes, the Normalized Difference Vegetation Index (NDVI_s_) [16], the Chlorophyll Normalized Difference Index (ChlNDI) [45], and the modified Simple Ratio 705 (mSR_705_) [46] were calculated using narrow-band reflectance values as follows:

$${\text{NDVI}}_{\text{s}}=\frac{\left({\text{R}}_{900}-{\text{R}}_{680}\right)}{\left({\text{R}}_{900}+{\text{R}}_{680}\right)}$$

$$\text{ChlNDI}=\frac{\left({\text{R}}_{750}-{\text{R}}_{705}\right)}{\left({\text{R}}_{750}+{\text{R}}_{705}\right)}$$

$${\text{mSR}}_{705}=\frac{\left({\text{R}}_{750}-{\text{R}}_{445}\right)}{\left({\text{R}}_{705}-{\text{R}}_{445}\right)}$$

where *R* indicates reflectance and the subindices indicate the wavelength in nm.

### Statistical analyses

Data were analysed using the SPSS 22.0 statistical package (SPSS Inc., Chicago, IL, USA). A bivariate correlation procedure was used to calculate Pearson correlation coefficients between vegetation indices and canopy biophysical (fIPAR and CCI) and agronomic variables (GY and aboveground biomass). Analyses of variance (ANOVA) were performed using a general linear model repeated-measures procedure to test the effects of both N fertilization treatments (between-subjects) and date of sampling (within-subjects) on the variables studied. Mean comparisons for treatment (N) and sampling date effects were performed using Student-Knewman-Keuls significant difference test (SNK). In order to evaluate the temporal stability of the relationships between vegetation indices and canopy biophysical variables (i.e., fIPAR and CCI), the general linear model repeated-measures procedure was used to perform a series of covariance analysis to test the effects of sampling date (i.e., phenological stage) on the dependence of vegetation indices on canopy biophysical variables (covariates). Differences among sampling dates were determined using the SIDAK test.

## Results

### Relationships between canopy biophysical variables and vegetation indices

**Fig 2:**
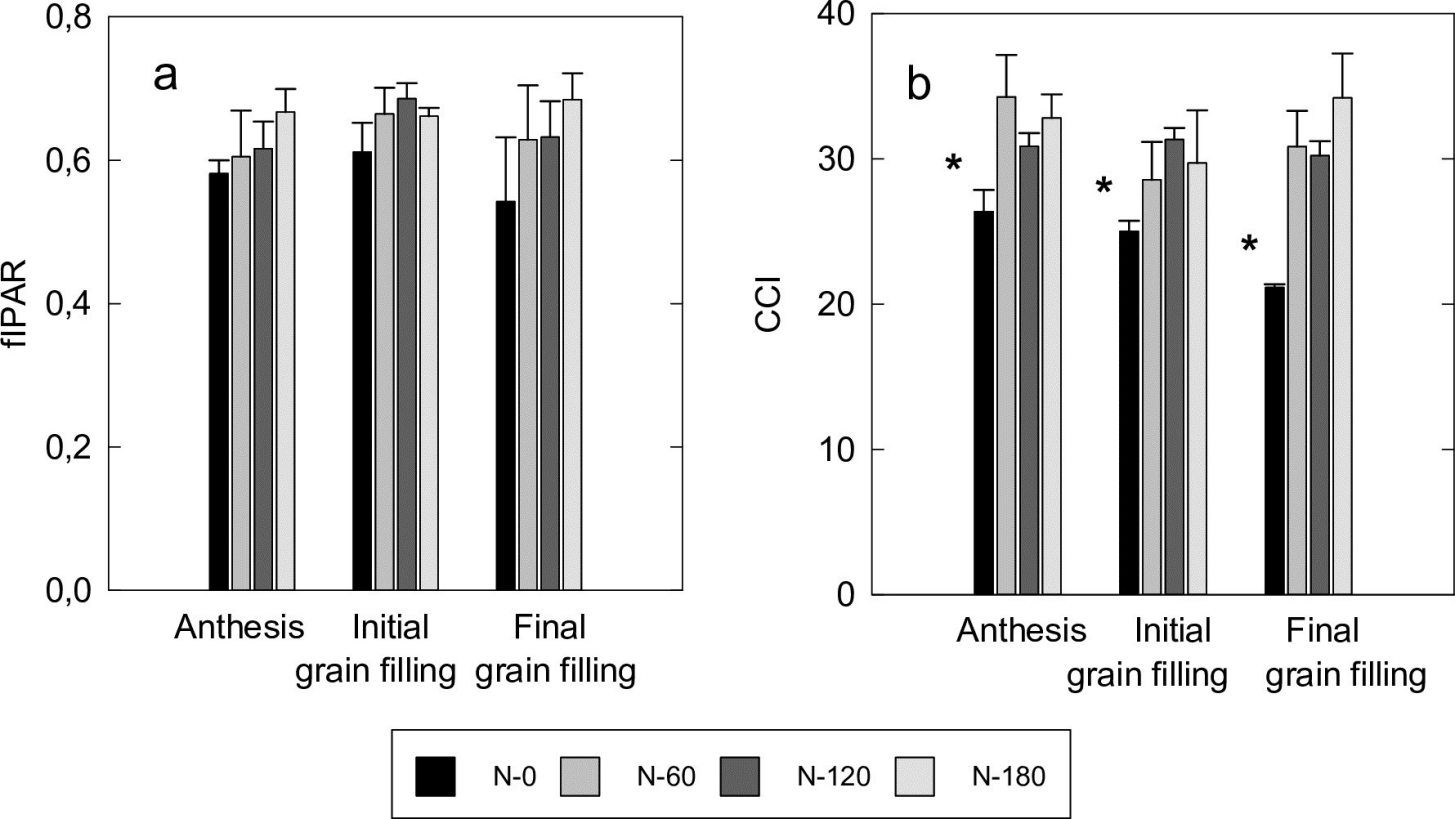
(a) Fractional Intercepted Photosynthetic Active Radiation (fIPAR) and (b) leaf Chlorophyll Content Index (CCI) at different phenological stages. Data are average values for each treatment and bars represent the standard error of the mean (n = 3). Columns marked with * significantly differ at *P* <0.05 within a phenological stage according to SNK.

Crop growth and development led to minor variation in fIPAR among N-treatments at the different phenological stages (i.e., anthesis, and initial and final grain filling). Indeed, fIPAR did not show significant variation (*P* < 0.05) across phenological stages (i.e., anthesis, and initial and final grain filling) neither among N-treatments (Fig 2) with average values ranging from 0.62 ± 0.02 (average ± standard error of the mean) to 0.66 ± 0.02 from anthesis to final grain filling. Contrastingly, CCI values showed significant (*P* < 0.05) variation across phenological stages and were significantly (*P* < 0.05) lower in non-fertilized (N-0) than in fertilized treatments. Differences in CCI between non-fertilized and fertilized treatments were larger at anthesis than at initial grain filling and increased again at the stage of final grain filling, when the canopy commenced to senesce. In addition, there was a significant correlation between fIPAR and chlorophyll content (CCI) with r values of 0.67, 0.75 and 0.66 (*P* < 0.05) at anthesis, initial and final grain filling stages, respectively.

Vegetation indices derived from the combination of visible (RGB) and near-infrared (NIR) bands (i.e. NDVI; SR, GNDVI and GSR) showed significant correlation with canopy biophysical variables (fIPAR and CCI) (Table 2). These correlations were higher with fIPAR than for CCI at anthesis and initial grain filling whereas at final grain filling (when the canopy started to senesce) the correlation coefficients among RGB-NIR based indices were slightly higher with CCI than with fIPAR.

**Table 2:** Correlation coefficients between fractional Intercepted PAR (fIPAR) and Chlorophyll Content Index (CCI) and vegetation indices (VI) obtained with a consumer-grade camera (see Table 1 for details on the vegetation indices studied) at each phenological stage (n = 12, except at initial grain filling where n = 10).

		Anthesis		Initial grain filling		Final grain filling	
Bands	VI	fIPAR	CCI	fIPAR	CCI	fIPAR	CCI
NIR / R	NDVI	0.82^**^	0.74^**^	0.73^*^	0.66^*^	0.78^**^	0.84^**^
	SR	0.82^**^	0.75^**^	0.69^*^	0.67^*^	0.77^**^	0.83^**^
NIR / G	GNDVI	0.83^**^	0.73^**^	0.70^*^	0.68^*^	0.83^**^	0.83^**^
	GSR	0.83^**^	0.74^**^	0.67^*^	0.69^*^	0.82^**^	0.83^**^
B / R	BR	0.70^*^	0.80^**^	0.69^*^	0.79^**^	0.31	0.73^**^
B / G	GB	-0.40	-0.60^*^	-0.09	-0.54	0.32	-0.25
G / R	GR	0.73^**^	0.71^**^	0.74^*^	0.56	0.61^*^	0.78^**^
	NDVIg	0.73^**^	0.70^*^	0.75^*^	0.57	0.60^*^	0.79^**^
	SAVIg	0.21	0.28	0.64^*^	0.23	0.57	0.66^*^
	OSAVIg	0.60^*^	0.61^*^	0.77^**^	0.50	0.62^*^	0.78^**^
R / G / B	VARI	0.75^**^	0.80^**^	0.76^*^	0.70^*^	0.50	0.79^**^
	NDVIrgb	0.74^**^	0.79^**^	0.76^*^	0.71^*^	0.46	0.79^**^
	SRrgb	0.75^**^	0.80^**^	0.75^*^	0.70^*^	0.47	0.78^**^

* and ** indicate significant correlation at *P* <0.05 and *P* < 0.01, respectively

Vegetation indices calculated using two visible bands were related to fIPAR and CCI in a variable degree. Among these indices, the ratio BR outperformed other indices at estimating chlorophyll content (CCI) across phenological stages with r values ranging from 0.73 to 0.80 (*P* < 0.01). Indices formulated using green and red bands, GR, NDVIg and OSAVIg showed significant correlation (*P* < 0.01) with CCI at anthesis and final grain filling, whereas the correlation between the indices GR and NDVIg with CCI at the initial grain filling stage was only marginally significant (r = 0.56, *P* < 0.10). In addition, the indices GR, NDVIg and OSAVIg showed significant correlation with fIPAR (i.e.,) at each sampling stage with r values ranging from 0.60 (*P* < 0.05) to 0.77 (*P* < 0.01).

Additionally, vegetation indices formulated using three visible bands (SRrgb, NDVIrgb and VARI) showed significant correlation against both CCI and fIPAR, except at the final grain filling stage when the correlation between these indices and fIPAR was not significant.

**Fig 3:**
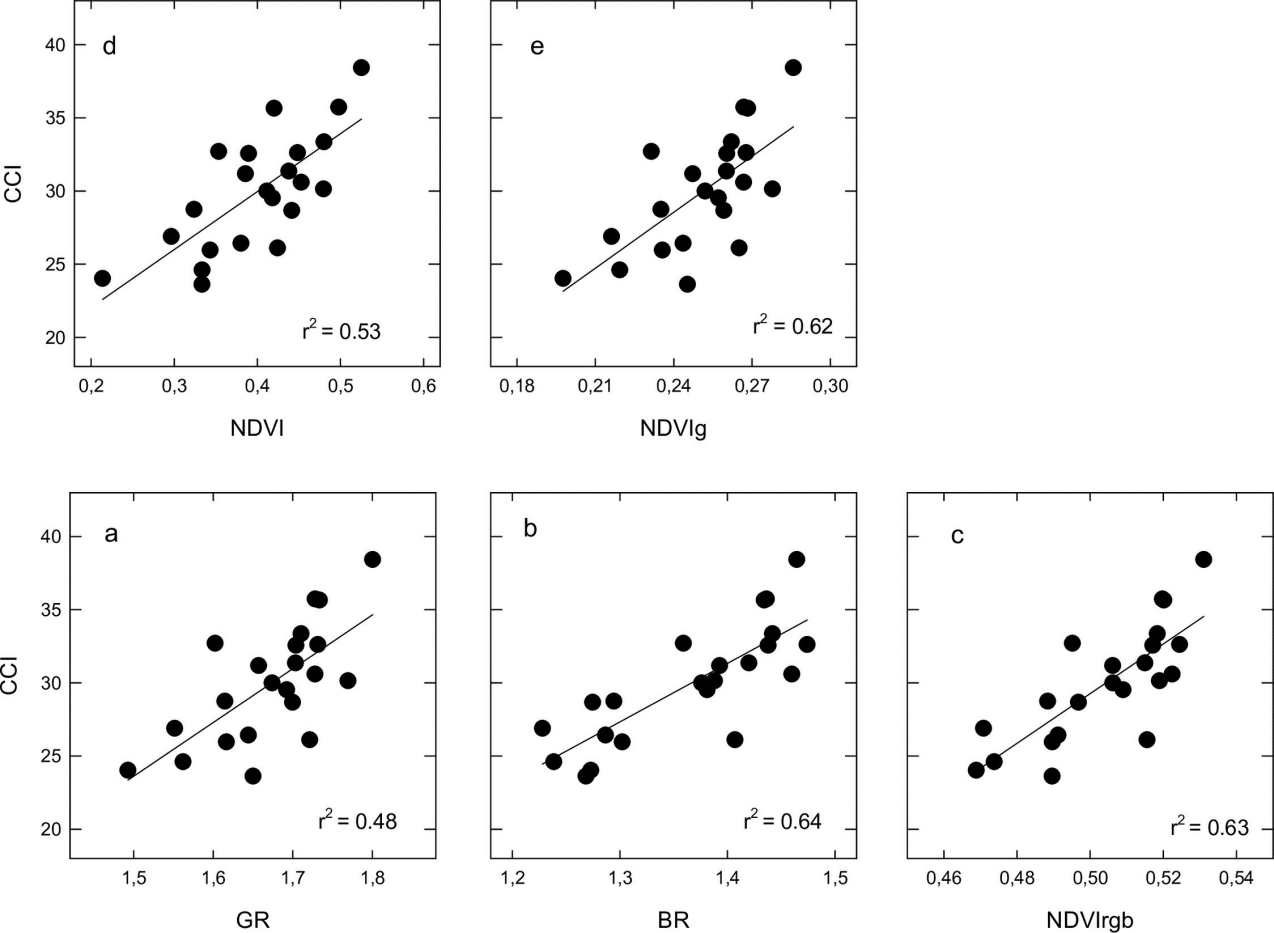
Relationships between camera derived vegetation indices (a) GR, (b) BR (c) NDVIrgb, (d) NDVI and (e) NDVIg (see Table 1 for a definition if each index) and the Chlorophyll Content Index (CCI). Pooled data for sampling dates corresponding to flowering and initial grain filling (n = 22).

We conducted a series of covariance analyses in order to evaluate the temporal stability of the relationships between the vegetation indices BR, GR, NDVI, NDVIg and NDVIrgb and the canopy biophysical variables CCI and fIPAR. The results showed that the dependence of vegetation indices on canopy variables was significantly (*P* < 0.01) affected by sampling date (i.e. phenological stage). Thus, the relationships between either GR or NDVIg vs. fIPAR showed no significant difference between initial and final grain filling stages but differed significantly (*P* < 0.01) at anthesis. In addition, the relationships between either BR and NDVIrgb and fIPAR significantly differed between anthesis and final grain filling whereas the relationships at initial grain filling did not differ from those at anthesis or final grain filling. Finally, the relationships between NDVI and fIPAR were found to be significantly different (*P* < 0.01) at each phenological stage (see S1 Fig). Similarly, there were distinct relationships (*P* < 0.01) between vegetation indices and CCI among phenological stages. However, all the indices studied (i.e. BR, GR, NDVI, NDVIg and NDVIrgb) showed a similar response. Thus, the relationships between vegetation indices and CCI were similar at anthesis and initial grain filling stages but significantly differ (*P* < 0.05) at the final grain filling stage. The relationships between vegetation indices and CCI at anthesis and initial grain filling stages (pooled data) are shown in Fig 3. Among the indices formulated with visible bands, BR and NDVIrgb, which included a blue band in their formulation, showed the highest coefficient of determination with CCI (r^2^ ~ 0.64; *P* < 0.01) followed by GR (r^2^ = 0.48; *P* < 0.01). In addition, the indices NDVI and NDVIg showed significant relationships against CCI with r^2^ = 0.53 and r^2^ = 0.62 (*P* < 0.01), respectively.

### Capacity to differentiate canopy traits related to N-status

To further assess the capability of vegetation indices at monitoring canopy variables related to crop N status, we evaluated the effects of N treatment and sampling date on the vegetation indices studied. There was no significant interaction between sampling date (i.e., phenological state) and N treatment for the indices studied. At anthesis, the indices NDVI, NDVIg and GR did not show significant differences among N treatments whereas NDVIrgb and BR showed significant differences (*P* < 0.05) between control (fertilizer not added, N-0) and fertilized treatments (Table 3). Subsequently, at initial grain filling, all the vegetation indices studied showed a dose-dependent response, with significant differences between control and fertilized treatments except for NDVI that did not show significant differences among treatments. Contrastingly, at final grain filling, all the vegetation indices studied, including NDVI, showed significant differences between control and fertilized treatments.

**Table 3:** Effects of N treatments on canopy fractional intercepted PAR (fIPAR) and leaf chlorophyll content index (CCI) and the vegetation indices NDVI, NDVIg, NDVIrgb, BR and GR (see Table 1 for details on the vegetation indices studied). Values are average (standard error of the mean) of 3 plots. Values followed by the same letter are not significantly different at *P* < 0.05 according to SNK.

N (Kg ha^-1^)	fIPAR	CCI	NDVI	NDVIg	NDVIrgb	BR	GR
Anthesis							
0	0.58 (0.02) a	26.4 (1.48) a	0.36 (0.04) a	0.24 (0.01) a	0.49 (0.01) a	1.26 (0.01) a	1.63 (0.04) a
60	0.61 (0.06) a	34.3 (2.88) b	0.42 (0.06) a	0.26 (0.01) a	0.51 (0.01) b	1.40 (0.05) b	1.72 (0.05) a
120	0.62 (0.04) a	30.9 (0.89) b	0.42 (0.02) a	0.26 (0.01) a	0.52 (0.01) b	1.43 (0.02) b	1.71 (0.01) a
180	0.67 (0.03) a	32.8 (1.62) b	0.48 (0.01) a	0.27 (0.01) a	0.52 (0.01) b	1.43 (0.02) b	1.74 (0.01) a
Initial grain filling							
0	0.61 (0.04) a	25 (0.73) a	0.31 (0.05) a	0.22 (0.01) a	0.48 (0.01) a	1.27 (0.01) a	1.57 (0.04) a
60	0.66 (0.04) a	28.6 (2.61) ab	0.36 (0.02) a	0.24 (0.01) ab	0.50 (0.01) b	1.35 (0.05) b	1.64 (0.02) ab
120	0.69 (0.02) a	31.3 (0.78) b	0.40 (0.02) a	0.25 (0.01) ab	0.51 (0.01) b	1.39 (0.02) b	1.66 (0.03) ab
180	0.66 (0.01) a	29.7 (3.62) ab	0.45 (0.03) a	0.26 (0) b	0.52 (0.01) b	1.42 (0.02) b	1.72 (0.01) b
Final grain filling							
0	0.54 (0.09) a	21.2 (0.21) a	0.23 (0.02) a	0.20 (0.01) a	0.46 (0.01) a	1.19 (0.03) a	1.51 (0.02) a
60	0.63 (0.08) a	30.9 (2.47) b	0.31 (0.01) b	0.23 (0.01) b	0.48 (0.01) b	1.26 (0.02) ab	1.61 (0.01) b
120	0.63 (0.05) a	30.2 (1.00) b	0.31 (0.03) b	0.23 (0.01) b	0.48 (0.01) b	1.29 (0.04) b	1.59 (0.04) b
180	0.68 (0.04) a	34.2 (3.05) b	0.35 (0.01) b	0.24 (0.01) b	0.5 (0.01) b	1.32 (0.02) b	1.64 (0.01) b

### Capacity to predict biomass and grain yield at harvest

Biomass at harvest ranged from 5431 ± 721 kg ha^-1^ (average ± sem) in N-0 treatment to 10243 ± 417 kg ha^-1^ in N-120 treatment whereas GY varied from 3254 ± 334 kg ha^-1^ to 5726 ± 236 kg ha^-1^. Aboveground biomass and GY showed significant differences (*P* < 0.05) among N-treatments, with lower values in unfertilized treatments (N-0) than in fertilized treatments (Table 4). There was a close correlation between biomass and GY (r = 0.98, *P* < 0.001). In addition, biomass and GY showed significant correlation with fIPAR (r = 0.73 and r = 0.72, *P* < 0.01, for biomass and GY, respectively) and CCI (r = 0.62 and r = 0.61, *P* < 0.01, for biomass and GY, respectively).

**Table 4:** Wheat grain yield and aboveground biomass (Biomass) as affected by N fertilization. Values are the average (standard error of the mean) (n = 3). Values followed by different letters differ significantly (p < 0.05) according to SNK.

N treatment	Biomass (Kg ha^-1^)	Grain yield (Kg ha^-1^)
N-0	8217 (1768) a	4523 (820) a
N-60	10612 (1577) b	5991 (1058) b
N-120	11113 (1022) b	6234 (578) b
N-180	10737 (811) b	6111 (403) b

Aboveground biomass and GY were consistently related to NDVI across sampling stages (*P* < 0.01) with average r values of 0.79 and 0.81, for biomass and GY, respectively (Table 5). Contrastingly, NDVIg and GR showed variable correlation, although significant, against biomass and GY at the different phenological stages, with higher values at initial grain filling (r values ranging from 0.80 to 0.84, *P* < 0.01) than at anthesis or final grain filling (r values ranging from 0.60 to 0.70, *P* < 0.05). Similarly, NDVIrgb and BR showed variable correlation against biomass and GY at the different phenological stages. These correlations were significant at anthesis and initial grain filling (r values ranging from 0.67 to 0.83, *P* < 0.05) but were not significant at final grain filling.

**Table 5:** Correlation coefficients between aboveground biomass (Biomass) and grain yield (GY) at harvest and vegetation indices at each phenological stage (n = 12, except at initial grain filling where n = 10).

		NDVI	NDVIg	NDVIrgb	BR	GR
Anthesis						
Biomass		0.73**	0.62*	0.73**	0.76**	0.63*
GY		0.78**	0.69*	0.78**	0.80**	0.70*
Initial grain filling						
Biomass		0.87**	0.81**	0.78**	0.67*	0.80**
GY		0.87**	0.84**	0.83**	0.73*	0.83**
Final grain filling						
Biomass		0.77**	0.60*	0.50ns	0.38ns	0.60*
GY		0.79**	0.65*	0.54ns	0.40ns	0.65*

* and ** indicate significant relationships at *P* < 0.05 and *P* < 0.01, respectively; and ns not significant

### Comparative performance camera vs spectroradiometer

**Fig 4:**
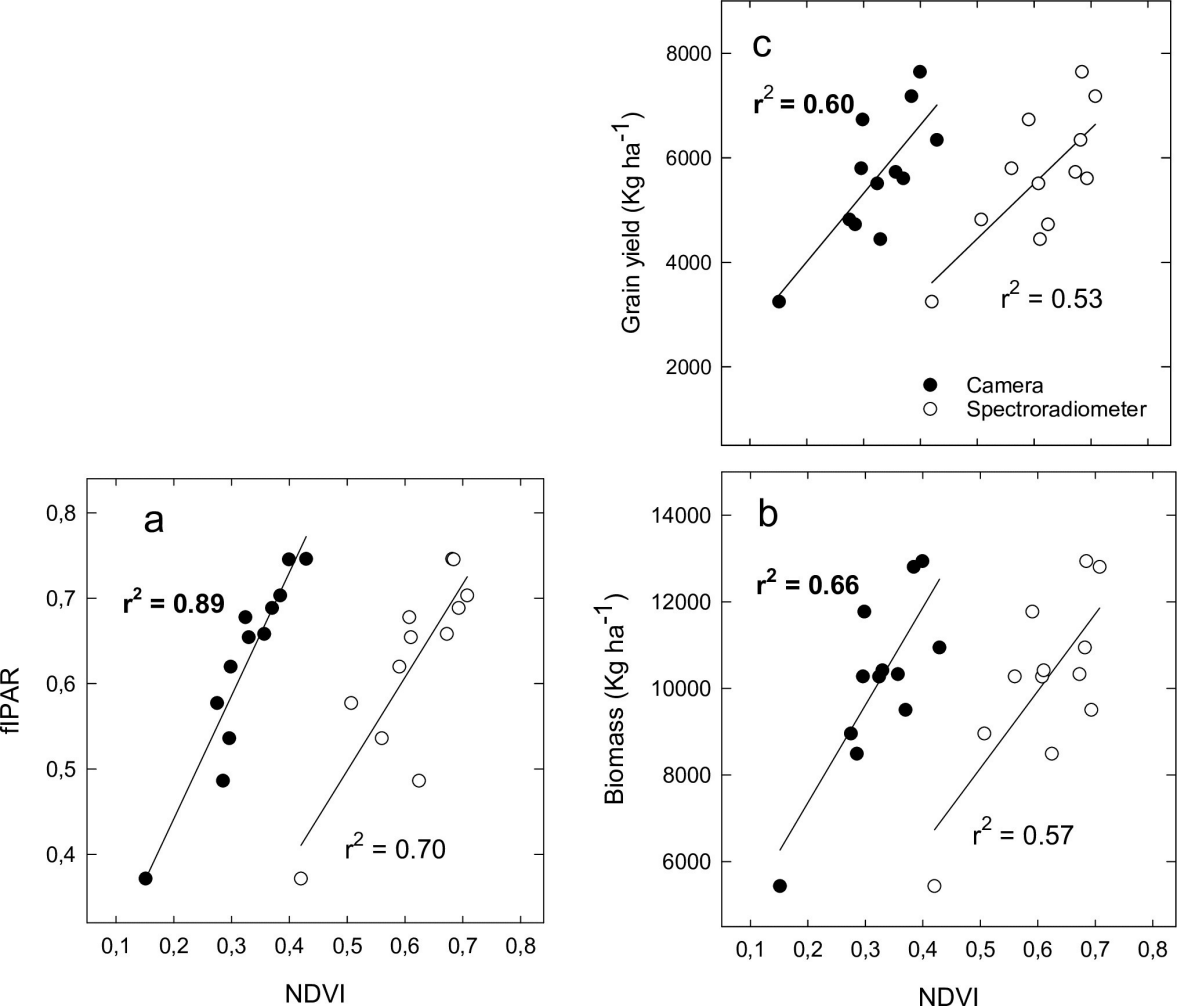
Relationships between (a) fractional Intercepted PAR (fIPAR), and (b) aboveground biomass (Biomass) and (c) grain yield at harvest with the Normalized Difference Vegetation Index (NDVI) derived either from a spectroradiometer or a conventional camera at final grain filling (n = 12).

**Fig 5:**
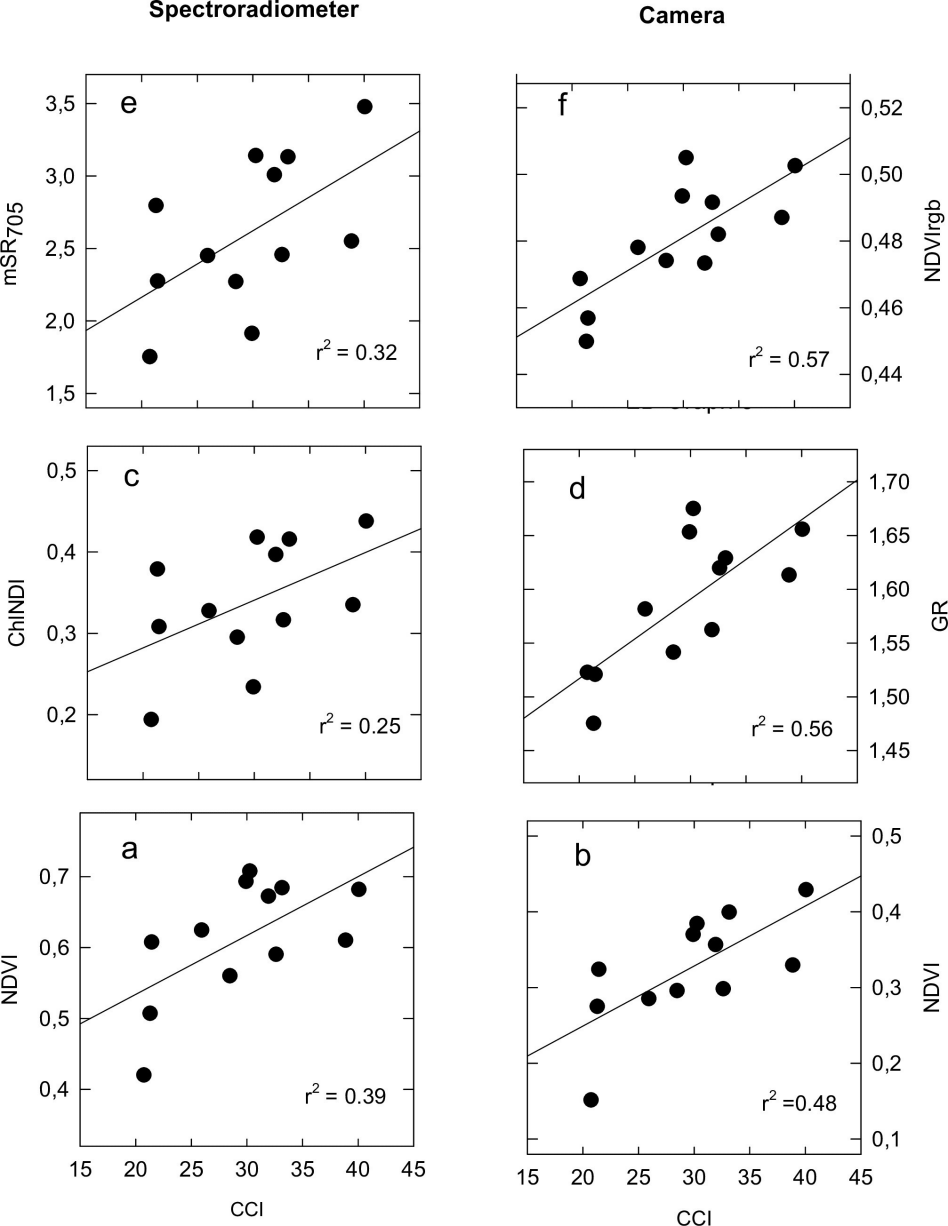
Relationships between the spectral indices (a) NDVI, (c) ChlNDI and (e) mSR_705_ derived from a spectroradiometer or (b) NDVI, (d) GR and (f) NDVIrgb obtained with a conventional camera with Chlorophyll Content Index (CCI). Data are for final grain filling (n = 12).

At final grain filling the capability of spectral indices calculated using a conventional camera at estimating canopy variables was compared to that of indices obtained using a scientific-grade spectroradiometer. The relationships between NDVI derived from a conventional camera against fIPAR, biomass and GY were higher than those derived from the NDVI obtained with the spectroradiometer (Fig 4). In addition, NDVI and red edge indices (R_750_/R_705_ and ChlNDI) obtained with the spectroradiometer showed significant relationships (r^2^ values ranging from 0.25 to 0.39; *P* < 0.05) with CCI (Fig 5). Nonetheless, indices calculated using a conventional camera related to CCI in a larger degree (r^2^ values ranging from 0.48 to 0.57; *P* < 0.01) than those derived from spectroradiometer data (Fig 5).

## Discussion

Remote sensing of crop N status is feasible in the visible spectral bands due to the close association between N and chlorophyll content [47], which largely determines the spectral signatures in this region. Previous studies have explored the capability of vegetation indices derived from commercial-grade cameras (RGB) at estimating either N or chlorophyll content. In wheat, indices based on green and red bands have been related to N content [28, 29]. Similarly, in our study, ratio and normalized difference indices based on green and red bands (i.e., GR and NDVIg) were found to be closely related to chlorophyll content. Although the NDVIg was designed to provide estimates of canopy cover from sparse canopies [43], our results suggest that NDVIg might be also a good estimator of chlorophyll content. In our study, data were acquired at stages where the soil contribution was minor -for the maximum LAI in wheat under Mediterranean conditions occurs at booting- which might be the reason for the observed similar capability at estimating chlorophyll content between soil adjusted indices aimed to correct the effects of varying canopy cover (SAVIg and OSAVIg) and either GR or NDVIg. However, the correlation between either GR or NDVIg and chlorophyll content was found to be variable at the different phenological stages. In wheat, Schirrman et al. [31] reported significant correlation between the ratio of green and red bands and N content at anthesis but no relationship at grain filling, as observed in our study at early grain filling. Nonetheless, in our study, indices derived from red and green bands showed significant correlation against chlorophyll content at later stages as has been previously reported [48].

In addition to decreases in chlorophyll content, N deficient plants usually develop higher concentrations of carotenoids and changes in these pigments might be captured combining blue and red bands. In agreement with previous studies in wheat [20], the blue to red ratio (BR) was found to be significantly related with chlorophyll content at the different developmental stages. Contrastingly, Schirrman et al. [31] did not find significant correlation between BR and tissue total N content whereas BR captured variation in biomass and LAI. In addition, in our study, GB showed poor capability at assessing variations in chlorophyll content whereas other studies have reported a close relationship between the narrow band blue green ratio and leaf N content on corn [49].

Hunt et al. [50] developed the triangular greenness index (TGI) based on the red, green and blue reflectance spectral features of chlorophyll and found it consistently related with plot-averaged chlorophyll-meter values at different spectral resolutions, including digital cameras. In the present study, vegetation indices calculated using three visible bands (VARI, SRrgb and NDVIrgb) outperformed the capacity of vegetation indices formulated using green and red bands at estimating chlorophyll content with correlation coefficients similar to that reported by Hunt et al. [50] in corn for VARI. Thus, it appears that the combination of RGB bands closely mirrors the relative changes in pigment composition.

Contrasting results regarding the capability of RGB based indices at estimating either N or chlorophyll content might be attributed to different band sensitivity to changes in pigment content. Filella et al. [20] reported higher sensitivity at the canopy level for G rather than R bands at increasing chlorophyll content, although both G and R reflectance saturated at high chlorophyll levels. Additionally, differences in crop status at the timing of image acquisition related to growth (soil cover and LAI), phenology (canopy greenness) as well as to the incidence of other stresses, particularly water stress, might mask the crop spectral response to N status[49]. Finally, differences in band amplitude, light conditions and geometry of acquisition are known to introduce some variability in the relationships between vegetation indices and either chlorophyll and N content. In our study, data were corrected to account for varying light conditions at the time of acquisition using a reference panel which might have partially improved the capability of vegetation indices at assessing chlorophyll content [51].

Nitrogen availability not only promotes changes in chlorophyll content but also affects crop growth. Since NDVI relates to both LAI and chlorophyll concentration, NDVI measurements have been used to assess crop N status [16,35]. In the present study, NDVI and GNDVI derived from a modified consumer camera were closely related to both leaf chlorophyll content and fIPAR as has been previously reported [16, 27, 52, 53]. In addition, and in agreement with previous studies [30,35], RGB indices showed comparable capability at assessing fIPAR than NIR band based indices (i.e., NDVI and GNDVI) at anthesis and initial grain filling. However, at final grain filling, when the canopy started to senesce, 3-band RGB based indices failed to track variation in fIPAR whereas NDVI and GNDVI were found to be related to fIPAR. Vegetation indices formulated using NIR and red bands are known to adequately characterize fIPAR by green, photosynthetically active vegetation [16]. Since changes in both LAI and chlorophyll concentration contribute to variation in canopy greenness, the ability of NIR based indices–when compared to RGB indices- at estimating fIPAR at stages with incipient senescence might be ascribed to the responsiveness of near-infrared wavelengths to changes in LAI. Different contribution of both LAI and chlorophyll concentration to changes in canopy greenness might also account for the distinct relationships (as indicated by the covariance analyses) between vegetation indices and fIPAR observed in our study among developmental stages. Contrastingly, the relationships between vegetation indices and chlorophyll content did not differ at developmental stages before the onset of senescence, suggesting that there was little variation in LAI between anthesis and initial grain filling. Indeed, vegetation indices, such as the NDVI, fail to distinguish changes in soil cover and LAI from changes in vegetation colour [54], thus, preventing the remote estimation of chlorophyll content at different crop growth stages [20,32]. Similarly, the capability of RGB indices at assessing chlorophyll content is known to be affected at LAI values < 2 and by changes in vegetation cover [50]. Nonetheless, due to the large spatial resolution present in RGB images, the confounding effects of varying vegetation cover might be easily corrected using appropriate image analysis techniques [55]

Monitoring of canopy structure through the NDVI provides indirect estimations of wheat crop’s biomass and GY [16]. Previous studies have reported varying capability of NDVI at estimating wheat aboveground biomass and GY as a function of the phenological stage [52,53] whereas, in our study, NDVI derived from a modified consumer grade camera provided similar estimates of GY from anthesis to grain filling. At anthesis, indices that included a blue band in their formulation (NDVIrgb and BR) showed comparable capability at predicting both biomass and GY to that of NDVI [56] whereas NDVIg and GR showed consistent estimates of both biomass and GY at initial grain filling. Therefore, in our study, the ability of RGB indices at predicting biomass and GY was found to be affected by plant phenology, in agreement with previous studies [33].

The capability of the vegetation indices derived from consumer-grade camera images at assessing chlorophyll content and fIPAR was confirmed by the similarity in the relationships between these canopy variables and the reflectance indices obtained with the spectroradiometer at final grain filling. Knowledge of the spectral response of each channel captured with the conventional camera might have allowed a more accurate comparison of the performance of both instruments. Nonetheless, RGB-NIR vegetation indices derived from a modified conventional camera (i.e., NDVI and GNDVI) showed similar correlation against fIPAR than the NDVI obtained with a spectroradiometer. Moreover, RGB-NIR vegetation indices provided at least similar predictions of biomass and GY than the NDVI obtained with a spectroradiometer. In addition, RGB based indices (i.e. GR, BR and NDVIrgb) showed even higher capability at assessing chlorophyll content than the ChlNDI index and the red-edge chlorophyll index (i.e. R_750_/R_705_), two widely accepted indices for assessing chlorophyll content.

Previous studies have demonstrated the capability of indices formulated using visible bands (i.e., RGB indices) at distinguishing between N fertilization treatments (non-fertilized vs. fertilized) [50]. In our study, at anthesis, leaf chlorophyll content was significantly lower in non-fertilized than in fertilized treatments. In accordance with these observed differences in leaf chlorophyll content, NDVIrgb and BR showed significant differences between unfertilized and fertilized treatments whereas indices that combined G and R bands, as well as NIR based indices, failed to track differences in chlorophyll content, as previously reported in corn [30]. Thus, in our study, NDVIrgb and BR were as reliable as a reference instrument for crop N-management at assessing chlorophyll content at anthesis, a growth stage when N fertilization can still have an effect on grain protein content [6]. In addition, differences in NDVIrgb and BR between unfertilized and fertilized plots were kept on the following developmental stages suggesting that these indices might potentially predict grain protein content [36]. The results herein reported are promising considering that these indices showed a dose-dependent response at the range of fertilizer rates used in this study. In our experiment, there were no differences in chlorophyll content among N fertilized levels -except when no nitrogen was applied-, thus, more studies are needed to evaluate the capability of RGB indices at discriminating areas of intermediate N status (i.e. chlorophyll content). In addition, future studies should also focus on earlier growth stages (i.e. from late tillering to early stem elongation) critical for N fertilization management in the field. In summary, our results show that consumer-grade camera based vegetation indices may have great potential for grain yield prediction and crop N status assessment. The development of low cost remote sensing techniques to assess crop N status with appropriate timing of acquisition might be a valuable tool to improve N fertilizer management in rain fed environments while increasing N use efficiency and reducing environmental impacts. In addition, in-season diagnosis of wheat N status might offer a sustainable and cost-effective opportunity to increase wheat GY and grain protein levels.

Further research is needed in order to confirm these results under a wide range of environmental conditions and N availabilities and should focus on diagnosing crop N status at critical developmental stages to support N-fertilizer management decisions. In addition, future studies should focus on the characterization of the camera’s spectral response and should develop methods to efficiently process and extract the information needed for practical applications [34].

## Conclusions

Vegetation indices based on RGB bands obtained with a standard consumer-grade camera provided reliable assessments of chlorophyll content and were related to fIPAR, aboveground biomass and GY at developmental stages prior to widespread senescence. In addition, RGB-NIR based indices derived from a modified conventional camera were able to capture variation in fIPAR and chlorophyll content. Moreover, RGB indices, particularly NDVIrgb and BR, provided chlorophyll content estimates comparable to those obtained with an optical reference instrument for crop N-management. Furthermore, vegetation indices based on consumer-grade cameras outperformed those derived from a scientific-grade spectroradiometer at estimating chlorophyll content as well as biomass and GY at harvest, supporting the use of consumer-grade cameras as simple and affordable tools for monitoring crop N-status and grain yield prediction. In summary, our results highlight the potential of standard and modified consumer-grade cameras as a low-cost remote sensing method for wheat N status assessment and grain yield prediction.

## Supporting information

S1 Fig Relationships between NDVI obtained with a modified consumer-grade camera and fractional Intercepted PAR (fIPAR) at each phenological stage (n = 12, except at initial grain filling where n = 10). (TIF)
