# Supplementary figures and images for: Use of consumer-grade cameras to assess wheat N status and grain yield
Source: PLoS One. 2019 Feb 15;14(2):e0211889. doi: 10.1371/journal.pone.0211889 (PMC6377115; doi:10.1371/journal.pone.0211889)

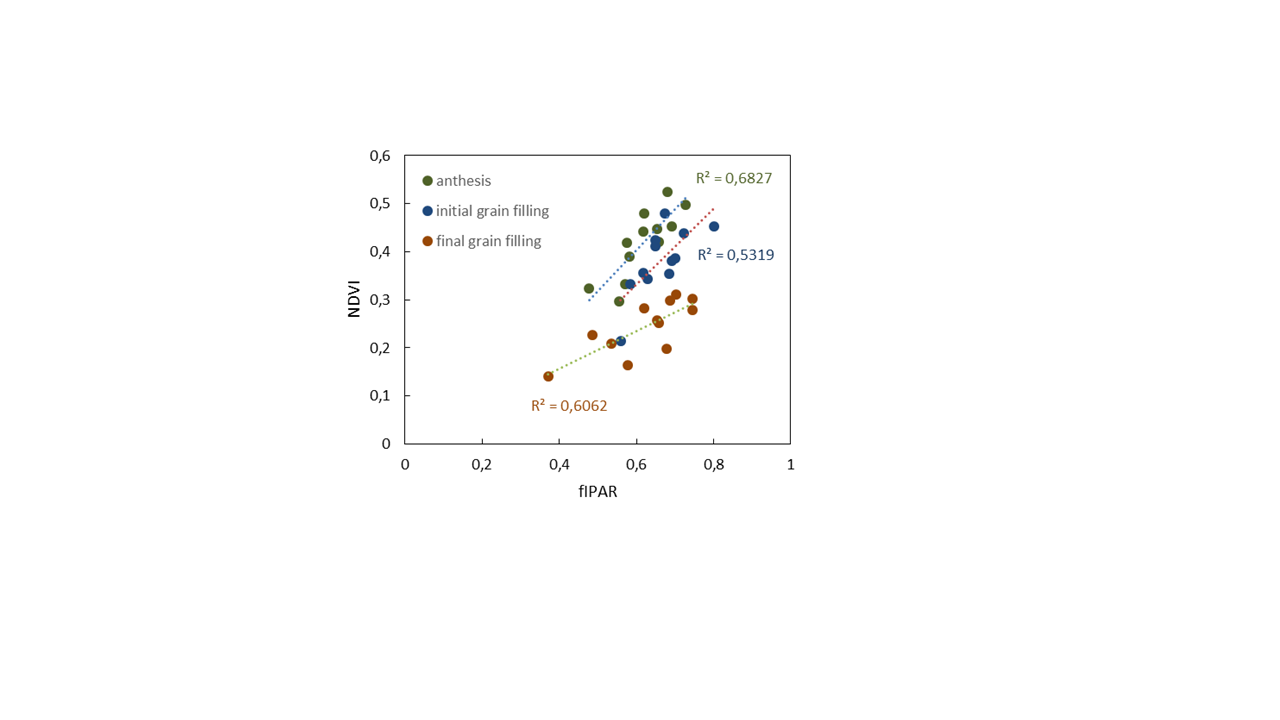

Supplement: S1 Fig — (TIF) [file pone.0211889.s001.tif]
